# Refreshed approaches to the therapy of malaria - the case of natural medicine

**DOI:** 10.1186/1475-2875-9-S2-O16

**Published:** 2010-10-20

**Authors:** Hagai Ginsburg

**Affiliations:** 1 Department of Biological Chemistry, Institute of Life Sciences, Hebrew University of Jerusalem, Jerusalem, Israel

## 

Although the completion of genome project of the most virulent malaria parasite *Plasmodium falciparum* in 2002 evoked great hopes for the identification of new targets for novel drugs, these have not yet been fulfilled. This disappointment and the successful use of natural compounds emerging from traditional medicine for the chemotherapy of malaria (quinine, artemisnin) have invigorated the search for new antimalarials from natural sources. Hundreds of thousands compounds were screened and some promising leads were identified.

However, the great advantage of traditional medicine is a result of demonstrable synergism between the constituents of the crude extracts. The origin and the modes of action of this synergism will be discussed. In conclusion, it is suggested that greater emphasis should be given to traditional medicine and some considerations for its controlled implementation will be mentioned.

